# Genetic divergence of farmed Atlantic halibut (*Hippoglossus hippoglossus*) and potential for impact on wild populations

**DOI:** 10.1186/s13104-026-07806-6

**Published:** 2026-04-10

**Authors:** Solveig Tronsgaard, María Quintela, Mikko Vihtakari, Geir Dahle, François Besnier, Ian Bradbury, Michael Møller Hansen, Kevin A. Glover, Per Erik Jorde

**Affiliations:** 1 Department of Biology - Genetics, Ecology and Evolution, Aarhus University, Aarhus, Denmark; 2 Institute of Marine Research, Group of Population Genetics, Bergen, Norway; 3 Institute of Marine Research, Group of Deepwater and Cartilaginous Fish, Tromsø, Norway; 4 Fisheries and Oceans Canada, Northwest Atlantic Fisheries Centre, 80 E White Hills Rd, St. John’s, NL A1C 5X1 Canada; 5 Biology Department, Dalhousie University, 6050 University Avenue, Halifax, NS B3H 4R2 Canada; 6 Institute of Marine Research, Group of Population Genetics, Flødevigen Research Station, His, Norway

**Keywords:** Atlantic halibut, *Hippoglossus hippoglossus*, Population structure, Aquaculture, SNPs, Effective population size

## Abstract

**Objective:**

Domestication of fish for aquaculture involves the production of strains that differ genetically from their wild counterparts. This is a concern when commercial production occurs in installations from which individuals can escape into the wild and thereafter interbreed with wild conspecifics. Using a panel of 96 SNPs, we compared genetic diversity within and among samples of Atlantic halibut collected from aquaculture strains and from the wild across the species’ distribution range in the North Atlantic.

**Results:**

All farmed samples differed significantly from the wild halibut samples and from each other. In contrast, no differences were found among the wild samples across the North Atlantic. Estimates of effective population size yielded low numbers (5.9 to 15.7) in all farmed strains, in contrast with infinite point estimates for all wild samples. The low estimates for farmed strains are consistent with extensive genetic drift as the primary cause of their divergence. We conclude that, because aquaculture-reared Atlantic halibut differ genetically from wild relatives, escapement of aquaculture-reared Atlantic halibut represents a potential threat to the genetic integrity of natural populations, should they interbreed in large numbers.

**Supplementary Information:**

The online version contains supplementary material available at 10.1186/s13104-026-07806-6.

## Introduction

Artificial rearing and production of aquatic organisms differ from most other forms of husbandry in that wild conspecifics are still extant and often present in the vicinity of the rearing facilities. While land-based facilities exist, most aquaculture of marine and anadromous fish takes place in open net pens in the ocean. This practice risks the escapement of farmed-reared fish into the natural environment, where they can interfere and potentially interbreed with natural conspecifics. The genetic impact of escaped hatchery fish on natural populations has been extensive for the widely bred Atlantic salmon e.g. [19], and the risk is apparent also for other aquaculture species e.g. [2, 28].

The Atlantic halibut (*Hippoglossus hippoglossus*) has been commercially reared in Norway for nearly three decades, producing annually between 3,000 and 4,000 metric tonnes (Norwegian Fisheries Directorate), approximately equal to the recent annual landings of the species in Norway [33]. While this volume is low compared to Atlantic salmon production (1.5 million tonnes), and the risks of escapement may be perceived as proportionally lower, escapes of large numbers (tens of thousands) of farmed Atlantic halibut have been reported (in 2017 and 2022, according to the Norwegian Directorate of Fisheries). Currently, no studies have genetically characterized strains of Atlantic halibut and compared them with wild conspecifics; therefore, the potential for genetic change in natural populations arising from such escapes remains unknown.

Here, we report a SNP panel genotyped at 11 samples (i.e., collection of individuals) collected from seven aquaculture farms in Norway and for samples of wild Atlantic halibut collected from the Northeast and Northwest Atlantic. The data were originally collected and screened at the IMR laboratory in Bergen, Norway, and analysed as part of Rasmussen [38] at Aarhus University, Denmark. The objective of the present study is to use these data to analyse the extent to which hatchery-reared Atlantic halibut differs genetically from natural populations and identify the causes of divergence. In addition, the potential consequences for natural populations, should escaped farmed halibut successfully interbreed with wild populations, are discussed.

## Methods

The 1368 individuals genotyped included 1133 of farmed origin, collected during 2008 from seven different farms, and 235 wild fish from the Gulf of Maine, Newfoundland, and the Norwegian coast, respectively (Table 1). DNA was extracted from fin clips and/or gill tissue preserved in ethanol 100% using HotSHOT Buffers [44].

**Table 1:** Sampling locations and summary genetic diversity statistics for Atlantic halibut genotyped at 96 polymorphic SNP loci

Type	Sample	*N*	Latitude	Longitude	H _S_	F _IS_	*N*_e_ (95% CI)*
Wild	GulfMaine_W	48	41.7° N	69.6° W	0.312	0.0131	∞ (436.9, ∞)
	Newfoundland_W	36	47.5° N	56.2° W	0.318	0.0485	∞ (∞, ∞)
	Norway69_71_W	40	70.0° N	19.0° E	0.305	-0.0140	∞ (∞, ∞)
	Lofoten_W	30	–	–	0.316	0.0304	∞ (1170.7, ∞)
	Norway_67_68_W	62	68.0° N	13.0° E	0.315	0.0285	∞ (∞, ∞)
	Norway_61_63_W	19	62.0° N	6.0° E	0.317	0.0341	∞ (79.6, ∞)
Farmed	BH_F	68	–	–	0.313	-0.0650	12.1 (10.8, 13.5)
	FH_F	185	–	–	0.314	0.0160	14.8 (13.7, 15.9)
	MH_F	112	–	–	0.326	-0.0772	12.1 (11, 13.2)
	NK_F	292	–	–	0.311	-0.0044	14.7 (13.7, 15.7)
	NS_F	80	–	–	0.307	-0.0204	15 (13.6, 16.5)
	SW1_F	43	-	–	0.308	0.0146	8.5 (7.3, 9.8)
	SW2_F	36	–	–	0.308	-0.0897	5.9 (4.2, 7.1)
	SW3_F	30	–	–	0.299	-0.0381	10.6 (9, 12.5)
	SW4_F	50	–	–	0.308	0.0022	14.8 (13, 16.8)
	SW5_F	38	–	–	0.232	-0.1899	9 (7.3, 10.9)
	TU_F	199	–	–	0.302	-0.0280	15.7 (14.5, 17)

*The LDNe method used to estimate effective population size lacks sufficient precision for estimating large population size when using relatively small samples. In such situations a negative number often results, implying that no genetic evidence for limited population size was detected. In consistency with common practice [48], we here report such negative estimates as “infinite” (∞), implicitly understood as “large”. Number of individuals per sample (N), geographic coordinates for the wild samples, expected heterozygosity (*H*_S_), inbreeding coefficient (*F*_IS_) and effective population size (*N*_e_: negative numbers interpreted as infinite, ∞) with 95% confidence interval. Farms are abbreviated as follows: Brandal Havbruk (BH), Fjord Halibut (FH), Marine Harvest (MH), Norsk Kveiteavl (NK), Nordic Seafarm (NS), Sterling White Halibut (SW) and Tubilah (TU). The names of the samples are followed by _W to depict samples of wild fish and by _F to identify the farmed ones. Geographic positions for wild individuals are the averages of the coordinates of the sampling stations (a dash means not available/applicable)

In 2019, reads of pool genome sequencing of female and male Atlantic halibut were mapped independently to a draft reference genome available at the time (Rolf Edvardsen, personal communication)— and later on published as Edvardsen et al., [10]— using the BWA *aln* function [31]. Polymorphic loci were detected using the SAMTOOLS function *mpileup* [32], and two sets of SNPs were selected. First, a set of randomly sampled SNPs from the available quality filtered loci, and secondly, SNPs displaying the largest allele frequency differences between males and females were kept as potential candidates for sex markers. In total, 202 SNPs were arranged into seven multiplexes using the Assay Designer option of the Typer v5 program (Agena Biosciences CA, USA). Primers were designed, and individuals were genotyped using the Sequenom MassARRAY iPLEX Platform as described by Gabriel et al., [16]. The number of SNPs retained for the present analyses was pruned based upon the following criteria: 23 SNPs with > 21% missing data were discarded, together with 53 monomorphic ones and 15 loci significantly departing from Hardy-Weinberg equilibrium. In addition, markers were mapped to the recently published genome assembly of the species [11] and filtered by removing 11 SNPs residing in the sex-associated region on Chr 12 and 4 SNPs that were at a distance of < 100 Kb from the closest one. The final dataset thus contained 1368 individuals genotyped at 96 polymorphic loci distributed across most of 24 chromosomes of the species (Supplement Fig. S1). Information about loci, multiplex reactions together with the raw data is publicly available (See Availability of Data and Materials).

To assess if those 96 SNPs would accurately discriminate between individuals in a population, the genotype accumulation curve was built using the function *genotype_curve* in the R (R Core [43]) package *poppr* [29]. Estimates of effective population size (*N*_e_) were obtained using the LD method implemented in LDNe v.1.31 [47] setting 0.05 as the lowest allele frequency and calculating parametric 95% confidence intervals around the point estimates. Because this technique tends to produce negative estimates for large populations with limited data, such estimates were interpreted and reported as “infinite” (cf. Waples and Do [48], . Different subsets of SNPs, representing different minimum distances along the genome, were evaluated when estimating *N*_e_. Genetic diversity or within-population heterozygosity (*H*_S_), inbreeding coefficient (*F*_IS_), and genetic divergence were estimated via pairwise *F*_ST_ [49] computed with Genepop v4.8.2 [39, 41]. Genepop was also used to test for genetic differentiation using the exact G test for pairs of samples and combined over loci with Fisher’s summation method. The resulting table of p-values was evaluated for significance using the FDR approach Benjamini and Yekutieli [4] implemented in the R function *p.adjust*. Finally, the relationship among individuals was examined using the Discriminant Analysis of Principal Components (DAPC) [27] implemented in the R package *adegenet* [25] with groups defined using geographically explicit locations. The cross-validation function [26, 34] was used to avoid overfitting.

Population structure was further investigated using the Bayesian clustering approach implemented in STRUCTURE v.2.3.4 [37] and conducted via ParallelStructure [6]. Analyses were performed under a model assuming admixture and correlated allele frequencies, both with and without incorporating sampling location as prior information, as the latter can increase sensitivity to subtle structure without biasing inference when structure is absent [23]. Three replicate runs were carried out for each value of K from 1 to 5, with a burn-in of 100,000 iterations followed by 1,000,000 Markov chain Monte Carlo (MCMC) iterations. For selected values of K, replicate runs were aligned and averaged using CLUMPP v.1.1.1 [24] under the FullSearch algorithm and the G’ pairwise matrix similarity statistic, and results were visualised as bar plots.

## Results

**Fig. 1:**
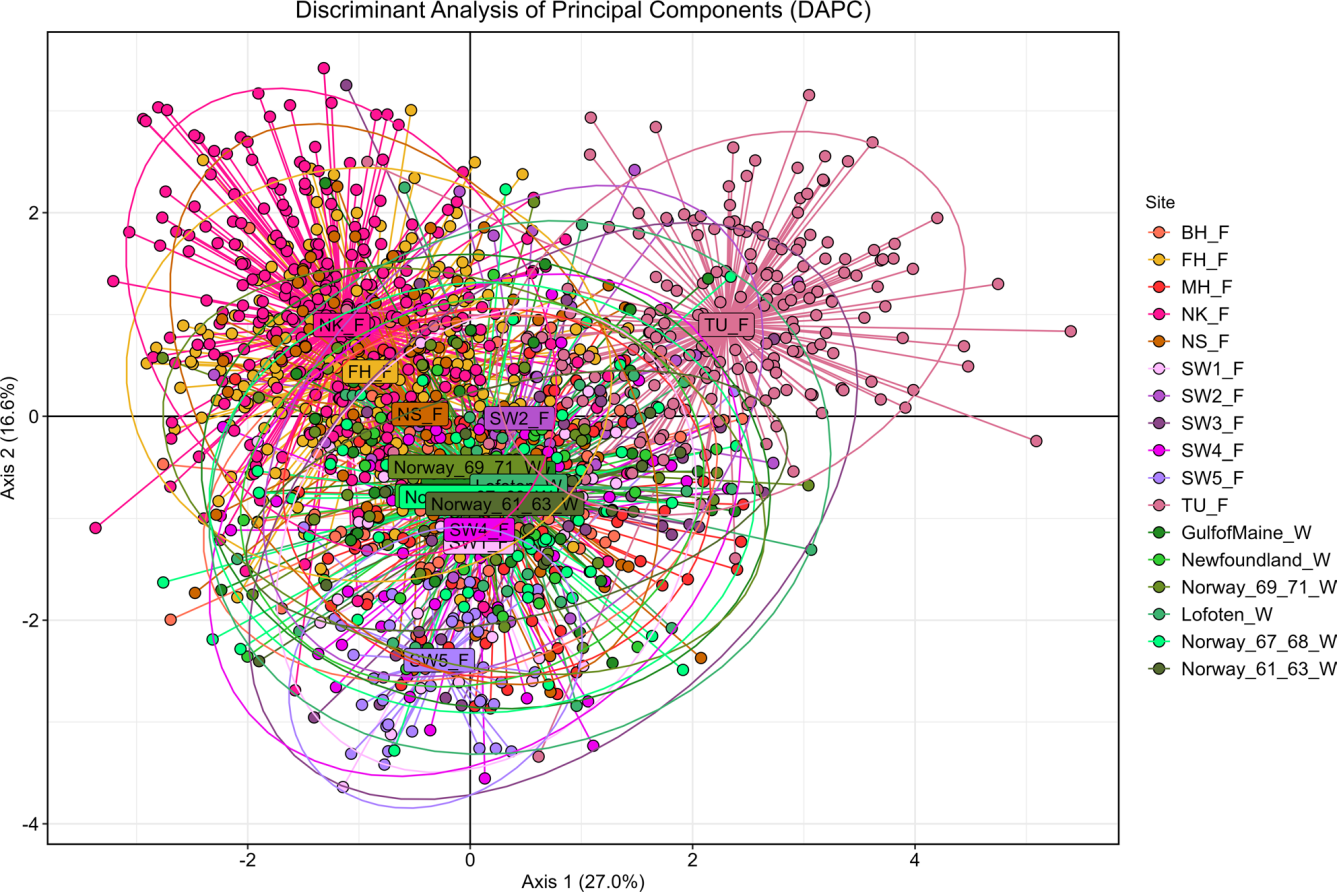
Discriminant analysis of principal components (DAPC) of Atlantic halibut based upon 96 polymorphic SNP loci. The axes correspond to the two first discriminant functions after retaining 60 principal components. Individuals from different geographically explicit samples are represented by coloured dots, and name labels are centred on the mean of the inertia ellipse. Greenish colours depict the wild individuals

The genotype accumulation curve reached a plateau with one third of the markers (Supplement Fig. S2), which reveals that the SNP panel used displayed enough power to discriminate between unique genotypes from the same sample. Genetic diversity (average heterozygosity: *H*_S_) was similar among samples but slightly lower in samples of farmed origin (averages 0.314 and 0.303 among wild and farmed, respectively). The estimated effective population size (*N*_e_) ranged from 5.9 to 15.7 in the farms with narrow confidence intervals (CI) (Table 1, based on 96 SNPs). The results were robust to different filtering of SNPs, representing different minimum distances along chromosomes (cf. Suppl. Fig. S3). In contrast, estimates were considerably higher in the wild populations, with all point estimates being infinite and lower 95% CI not overlapping farm estimates (Table 1). Pairwise *F*_ST_ (Table 2) revealed no significant differentiation among wild samples despite being collected across the North Atlantic, whereas highly significant differentiation was detected between samples from farms with a few exceptions (SW4_F vs. Norway_61_36_W and SW1_F vs. SW4_F). Pairwise *F*_ST_ values conducted with the 111 unfiltered SNPs were, on average, only slightly larger (0.0020) than the filtered ones, with which displayed strong correlation (correlation coefficient: 0.9905). The farmed sample SW5_F resulted in the largest *F*_ST_ difference when compared to other samples (0.064–0.184). The first axis of the DAPC (Fig. 1), accounting for 33.6% of the variation, primarily discriminated between farm TU_F and partially NK_F, with the latter contributing to the second axis (14.8%). The third axis (12.4%) discriminated SW5_F (graph not shown) in agreement with pairwise *F*_ST_. In DAPC conducted with unfiltered loci, PC1 displayed a slightly better discrimination of farmed samples MH_F, BH_F and FH_F (Supplement Fig. S4). The low genetic differentiation among wild samples was reflected in their extreme overlapping in the DAPC. STRUCTURE conducted with and without priors revealed the lack of differentiation among wild samples as well as the overlap between wild and farmed samples depicted in DAPC (Supplement Fig. S5) despite the significant differentiation detected via pairwise *F*_ST_.

**Table 2:**
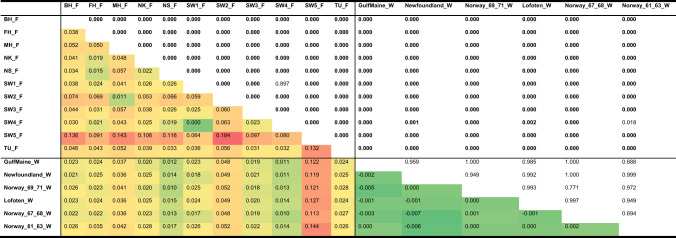
Genetic differentiation in Atlantic halibut

Negative *F*_ST_-values are interpreted as zero, i.e., there is no evidence for genetic difference in those cases. Heatmap of pairwise *F*_ST_ estimates across the 17 samples genotyped at 96 SNPs (below diagonal) and corresponding *P*-values above the diagonal, with the ones significantly different from zero after FDR correction highlighted in boldface type. Lines delimitate the farmed (_F) and wild (_W) origin of the individuals. Colour scale indicates the magnitude of *F*_ST_ (green = low differentiation; red = highest differentiation)

## Discussion

The genetic observations are broadly consistent with a scenario of little to no population genetic structure in wild Atlantic halibut, in contrast to highly significant differences among all samples taken from farms and between the farmed and wild samples. Genetic differentiation between farmed and wild conspecifics is highly common in fish, both when artificially reared fish are used to supplement weak wild populations [1] and between commercially farmed strains and wild conspecifics [2, 7, 19].

Although limited by modest spatial and genomic coverage, the apparent lack of pronounced genetic divergence among wild Atlantic halibut populations in the present study, even across large geographic distances, is consistent with earlier studies based on allozymes [14, 15], microsatellites [40], and SNPs [30], although the latter study did report on subtle but significant genome-wide environmentally associated structuring. Atlantic halibut bathypelagic eggs stay at 100–300 m depth, and although larvae have rarely been found in the wild [5, 21]; they are known to settle at the bottom some 90 days after hatching [21]. Altogether, this represents several weeks of eggs and larval drifting with currents such as the North Atlantic Current [22], and the corresponding opportunities for gene flow regionally. Unlike Greenland halibut [45], there is little information about trans-oceanic migrations in Atlantic halibut. Immature Atlantic halibut tend to stay in fjords and coastal areas during first 3–6 years of life [21], or conduct short migration in restricted geographic regions [20]; however, some can also perform relative long migrations crossing deep water areas in the North Atlantic [8, 21], which could also contribute to the mixing of geographically distant stocks as observed in this study. The weak genetic structure observed in the Atlantic halibut contrasts with findings in closely related Pleuronectidae species, such as the Greenland halibut and the Pacific halibut. These species exhibit subtle but statistically significant population structure across broad spatial scales in Greenland halibut [12, 51], as well as finer-scale genetic differentiation in both species [9, 17, 35]. Future studies employing higher-resolution genomic markers may reveal patterns of differentiation in Atlantic halibut not detected in the present study.

The main cause of divergence of farmed halibut strains both from natural populations and from each other is likely due to random genetic drift as reflected in the very small effective sizes estimated for the farmed strains (range 5.9 to 15.7). These effective numbers represent the immediate parent generation [46], with only minor input from earlier (presumed wild) generations, and imply that farmed strains were founded and maintained by very few individuals and/or that culture practices led to highly uneven parental contributions. In particular, the common practice of mixing sperm from several males to fertilize a batch of eggs has been shown to skew the representation of male parents through sperm competition [3, 36], thereby reducing *N*_e_ and increasing random genetic drift. It is also challenging to maintain large number of adult halibuts as broodstock, a problem that is likely to persist in coming generations and make it difficult to increase *N*_e_ substantially in the farms.

Low effective population sizes are expected to result in loss of genetic variation, as measured by average heterozygosity, of 1/2*N*_e_ per generation or 3.4 to 8.5% for our range of *N*_e_ estimates. The slight (non-significant) difference in heterozygosity (*H*_S_) between wild and farmed populations indicated an average loss of 3.6% in the samples from farms. This decline is thus within the range expected from our estimated *N*_e_ assuming a single generation of genetic drift. A single generation of drift is consistent with the farmed strains representing first-generation offspring of wild-caught parents, a common practice in halibut aquaculture at the time of sampling [18].

Genetic divergence and loss of genetic variation during this early stage of domestication (sampled in 2008) is relatively modest (perhaps excepting strain SW5_F) but is an ongoing evolutionary process as farmed strains adapt to farm conditions and imposed selective regimes. The low effective population sizes, unless substantially increased through changes in hatchery practice, will induce further loss of genetic variation over time and, in combination with selection, lead to increased diversification from natural populations. Loss of genetic variation and associated increase in inbreeding can be retarded by increasing broodstock sizes, by equalizing family sizes, e.g. through improving sperm mixing practices and/or culling large families, and by supplementing farmed broodstocks with wild-caught individuals. Because occasional events of large number of fish escaping from farms occasionally occur, there is an obvious risk of genetically depauperated escapees adversely affecting natural populations, should they successfully interbreed with wild fish [42]. Minimizing the number of escaped fish through improved net pen technology and operational procedures would be the most important preventive approach to avoid this adverse effect of aquaculture.

## Limitations

The present study was based on limited genomic and spatial coverage. None of our SNPs was located on chromosome 15, where a putative inversion associated with adaptive divergence has been reported [30]. This is a result of our SNP identification in 2019, when no chromosome-level reference genome was available; as a consequence, scaffolds representing chromosome 15 may have been accidentally missed. In this study we removed SNPs residing in the sex-associated part on chromosome 12 in order not to bias estimates of divergence if samples contained different numbers of individuals from opposing sexes e.g. [12, 13, 50], as phenotypic sex was not generally available. Low geographic coverage also limited our ability to detect genetic structuring among natural populations. Hence, the absence of evidence for population structuring in wild Atlantic halibut should not be overinterpreted. This study covers the early phase of commercial farming of Atlantic halibut, and the results may not reflect the current situation.

## Supplementary Information

Below is the link to the electronic supplementary material.

Supplementary Material 1.

## Data Availability

The pool genome sequencing data used for SNP mining is available via Edvardsen et al. (2022). The SNP raw data used in this study is publicly available through the repository of the Norwegian Research Information Repository at https://hdl.handle.net/11250/5318275 including also: SNPs plus flanking regions, primers for multiplex reactions, and graphic representation of SNPs mapped to the Atlantic halibut genome assembly.

## References

[1] Allendorf FW, Ryman N. Genetic management of hatchery stocks. In: Ryman N, Utter F, editors. Population Genetics and Fishery Management. Washington Sea Grant Program: University of Washington; 1987.

[2] Atalah J, Sánchez-Jerez P. Global assessment of ecological risks associated with farmed fish escapes. Global Ecol Conserv. 2020;21:e00842. 10.1016/j.gecco.2019.e00842.

[3] Bekkevold D, Hansen MM, Loeschcke V. Male reproductive competition in spawning aggregations of cod (*Gadus morhua*, L). Mol Ecol. 2002;11(1):91–102. 10.1046/j.0962-1083.2001.01424.x. 11903907 10.1046/j.0962-1083.2001.01424.x

[4] Benjamini Y, Yekutieli D. The control of the false discovery rate in multiple testing under dependency. Ann Stat. 2001;29:1165–88. 10.1214/aos/1013699998.

[5] Bergstad OA, Gordon JDM. First record of Atlantic halibut (*Hippoglossus hippoglossus* (L.)) larvae from the Skagerrak. ICES J Mar Sci. 1993;50(2):231–2. 10.1006/jmsc.1993.1025.

[6] Besnier F, Glover KA. ParallelStructure: a R package to distribute parallel runs of the population genetics program STRUCTURE on multi-core computers. PLoS ONE. 2013;8(7):e70651. 10.1371/journal.pone.0070651. 23923012 10.1371/journal.pone.0070651 PMC3726640

[7] Bolstad GH, Karlsson S, Hagen IJ, Fiske P, Urdal K, Sægrov H, Florø-Larsen B, et al. Introgression from farmed escapees affects the full life cycle of wild Atlantic salmon. Sci Adv. 2021;7(52):eabj3397. 10.1126/sciadv.abj3397. 34936452 10.1126/sciadv.abj3397 PMC8694624

[8] Bowering W. R. The distribution, age and growth and sexual maturity of Atlantic halibut (*Hippoglossus hippoglossus*) in the Newfoundland and Labrador Area of the northwest Atlantic. Canadian Technical Report of Fisheries and Aquatic Sciences, Newfoundland, Canada: Departement of Fisheries and Oceans, St. 1986John’s, 1986; 1432; 1–40. https://publications.gc.ca/collections/collection_2013/mpo-dfo/Fs97-6-1432-eng.pdf

[9] Drinan DP, Galindo HM, Loher T, Hauser L. Subtle genetic population structure in Pacific halibut *Hippoglossus stenolepis*. J Fish Biol. 2016;89(6):2571–94. 10.1111/jfb.13148. 27714808 10.1111/jfb.13148

[10] Edvardsen RB, Wallerman O, Furmanek T, Kleppe L, Jern P, Wallberg A, Kjærner-Semb E, et al. Heterochiasmy and the establishment of gsdf as a novel sex determining gene in Atlantic halibut. PLoS Genet. 2022;18(2):e1010011. 10.1371/journal.pgen.1010011. 35134055 10.1371/journal.pgen.1010011 PMC8824383

[11] Einfeldt AL, Kess T, Messmer A, Duffy S, Wringe BF, Fisher J, den Heyer C, et al. Chromosome level reference of Atlantic halibut *Hippoglossus hippoglossus* provides insight into the evolution of sexual determination systems. Mol Ecol Resour. 2021;21(5):1686–96. 10.1111/1755-0998.13369. 33655659 10.1111/1755-0998.13369

[12] Estévez-Barcia D, Roy D, Vihtakari M, Gíslason D, Lindegren M, Christensen A, Wheeland L, et al. Sex Influences the Genetic Structure of Greenland Halibut in the North Atlantic. Ecol Evol. 2025;15(2):e70822. 10.1002/ece3.70822. 39931248 10.1002/ece3.70822 PMC11810528

[13] Ferchaud AL, Mérot C, Normandeau E, Ragoussis J, Babin C, Djambazian H, Bérubé P, et al. Chromosome-level assembly reveals a putative Y-autosomal fusion in the sex determination system of the Greenland Halibut (*Reinhardtius hippoglossoide*s). G3 Genes|Genomes|Genetics. 2022;12(1). 10.1093/g3journal/jkab376. 10.1093/g3journal/jkab376 PMC8727965 34791178

[14] Fevolden SE, Haug T. Genetic population structure of Atlantic halibut, *Hippoglossus hippoglossus*. Can J Fish Aquat Sci. 1988;45:2–7.

[15] Foss A, Imsland AK, Nævdal G. Population genetic studies of the Atlantic halibut in the North Atlantic Ocean. J Fish Biol. 1998;53(4):901–5. 10.1111/j.1095-8649.1998.tb01845.x.

[16] Gabriel S, Ziaugra L, Tabbaa D. SNP genotyping using the Sequenom MassARRAY iPLEX Platform. Curr Protocols Hum Genet. 2009;60(1). 10.1002/0471142905.hg0212s60 10.1002/0471142905.hg0212s60 19170031

[17] Gíslason D, Estévez-Barcia D, Sveinsson S, Hansen A, Roy D, Treble M, Boje J, et al. Population structure discovered in juveniles of Greenland halibut (*Reinhardtius hippoglossoides* Walbaum, 1792). ICES J Mar Sci. 2023;80(4):889–96. 10.1093/icesjms/fsad011.

[18] Glover KA, Svåsand T, Olesen I, Rye M. (2006). Atlantic halibut - *Hippoglossus hippoglossus*. *Genetic impact of aquaculture activities on native populations. Genimpact final scientific report (EU contract RICA-CT-2005-022802), pp. 17–22.*

[19] Glover KA, Solberg MF, McGinnity P, Hindar K, Verspoor E, Coulson MW, Hansen MM, et al. Half a century of genetic interaction between farmed and wild Atlantic salmon: Status of knowledge and unanswered questions. Fish Fish. 2017;18(5):890–927. 10.1111/faf.12214.

[20] Godø OR, Haug T. Tagging and recapture of Atlantic halibut (*Hippoglossus hippoglossus)* in Norwegian waters. J Conseil. 1988;44(2):169–79. 10.1093/icesjms/44.2.169.

[21] Haug T. Biology of the Atlantic Halibut, *Hippoglossus hippoglossus* (L., 1758). In: Blaxter JHS, Southward AJ, editors. Advances in Marine Biology. Volume 26. Academic; 1990. pp. 1–70.

[22] Haug T, Kjørsvik E, Solemdal P. Vertical distribution of Atlantic halibut (*Hippoglossus hippoglossus*) eggs. Can J Fish Aquat Sci. 1984;41(5):798–804. 10.1139/f84-092.

[23] Hubisz MJ, Falush D, Stephens M, Pritchard JK. Inferring weak population structure with the assistance of sample group information. Mol Ecol Resour. 2009;9(5):1322–32. 10.1111/j.1755-0998.2009.02591.x. 21564903 10.1111/j.1755-0998.2009.02591.x PMC3518025

[24] Jakobsson M, Rosenberg NA. CLUMPP: A cluster matching and permutation program for dealing with label switching and multimodality in analysis of population structure. Bioinformatics. 2007;23(14):1801–6. 10.1093/bioinformatics/btm233. 17485429 10.1093/bioinformatics/btm233

[25] Jombart T. *adegenet*: a R package for the multivariate analysis of genetic markers. Bioinformatics. 2008;24:1403–5. 10.1093/bioinformatics/btn129. 18397895 10.1093/bioinformatics/btn129

[26] Jombart T, Collins C. A tutorial for discriminant analysis of principal components (DAPC) using adegenet 2.0.0. 2015; https://adegenet.r-forge.r-project.org/files/tutorial-dapc.pdf

[27] Jombart T, Devillard S, Balloux F. Discriminant analysis of principal components: a new method for the analysis of genetically structured populations. BMC Genet. 2010;11(1):94. 10.1186/1471-2156-11-94. 20950446 10.1186/1471-2156-11-94 PMC2973851

[28] Jorde PE, van der Meeren T, Quintela M, Dahle G, Mateos-Rivera A, Aase M, Norberg B, et al. Genetic analyses verify sexually mature escaped farmed Atlantic cod and farmed cod eggs in the natural environment. Evol Appl. 2024;17(4):e13688. 10.1111/eva.13688. 38633132 10.1111/eva.13688 PMC11022607

[29] Kamvar ZN, Tabima JF, Grünwald NJ. Poppr: an R package for genetic analysis of populations with clonal, partially clonal, and/or sexual reproduction. PeerJ. 2014;2:e281. 10.7717/peerj.281. 24688859 10.7717/peerj.281 PMC3961149

[30] Kess T, Einfeldt AL, Wringe B, Lehnert SJ, Layton KKS, McBride MC, Robert D, et al. A putative structural variant and environmental variation associated with genomic divergence across the Northwest Atlantic in Atlantic Halibut. ICES J Mar Sci. 2021;78(7):2371–84. 10.1093/icesjms/fsab061.

[31] Li H, Durbin R. Fast and accurate long-read alignment with Burrows–Wheeler transform. Bioinformatics. 2010;26(5):589–95. 10.1093/bioinformatics/btp698. 20080505 10.1093/bioinformatics/btp698 PMC2828108

[32] Li H, Handsaker B, Wysoker A, Fennell T, Ruan J, Homer N, Marth G, et al. The Sequence Alignment/Map format and SAMtools. Bioinformatics. 2009;25(16):2078–9. 10.1093/bioinformatics/btp352. 19505943 10.1093/bioinformatics/btp352 PMC2723002

[33] Lindgård EL, Berg E, Zimmermann F, Aschan M. Recent recovery and future prospects of the northeast Atlantic halibut stock. ICES J Mar Sci. 2025;82(9):fsaf166. 10.1093/icesjms/fsaf166.

[34] Miller JM, Cullingham CI, Peery RM. The influence of a priori grouping on inference of genetic clusters: Simulation study and literature review of the DAPC method. Heredity. 2020;125(5):269–80. 10.1038/s41437-020-0348-2. 32753664 10.1038/s41437-020-0348-2 PMC7553915

[35] Nielsen JL, Graziano SL, Seitz AC. Fine-scale population genetic structure in Alaskan Pacific halibut (*Hippoglossus stenolepis*). Conserv Genet. 2010;11(3):999–1012. 10.1007/s10592-009-9943-8.

[36] Ottesen OH, Babiak I, Dahle G. Sperm competition and fertilization success of Atlantic halibut (*Hippoglossus hippoglossus* L). Aquaculture. 2009;286(3):240–5. 10.1016/j.aquaculture.2008.09.018.

[37] Pritchard JK, Stephens M, Donnelly P. Inference of population structure using multilocus genotype data. Genetics. 2000;155(2):945–59. 10.1093/genetics/155.2.945. 10835412 10.1093/genetics/155.2.945 PMC1461096

[38] Rasmussen ST. Population structure of farmed and wild Atlantic halibut (*Hippoglossus hippoglossus*) and the potential for genetic assignment of farmed escapees. Department of Biology. Master: Aarhus University; 2020. p. 56.

[39] Raymond M, Rousset F. GENEPOP Version 1.2: Population genetics software for exact tests and ecumenicism. J Hered. 1995;86:248–9. 10.1093/oxfordjournals.jhered.a111573.

[40] Reid DP, Pongsomboon S, Jackson T, McGowan C, Murphy C, Martin-Robichaud D, Reith M. Microsatellite analysis indicates an absence of population structure among *Hippoglossus hippoglossus* in the north-west Atlantic. J Fish Biol. 2005;67(2):570–6. 10.1111/j.0022-1112.2005.00733.x.

[41] Rousset F. GENEPOP’007: A complete re-implementation of the genepop software for Windows and Linux. Mol Ecol Resour. 2008;8(1):103–6. 10.1111/j.1471-8286.2007.01931.x. 21585727 10.1111/j.1471-8286.2007.01931.x

[42] Ryman N, Laikre L. Effects of Supportive Breeding on the Genetically Effective Population Size. Conserv Biol. 1991;5(3):325–9. 10.1111/j.1523-1739.1991.tb00144.x.

[43] Team RC. (2025). R: A Language and Environment for Statistical Computing. R Foundation for Statistical Computing, Vienna, Austria. URL https://www.R-project.org/

[44] Truett GE, Heeger P, Mynatt RL, Truett AA, Walker JA, Warman ML. Preparation of PCR-quality mouse genomic DNA with hot sodium hydroxide and tris (HotSHOT). Biotechniques. 2000;29(1):52–4. 10.2144/00291bm09. 10907076 10.2144/00291bm09

[45] Vihtakari M, Elvarsson BÞ, Treble M, Nogueira A, Hedges K, Hussey NE, Wheeland L, et al. Migration patterns of Greenland halibut in the North Atlantic revealed by a compiled mark–recapture dataset. ICES J Mar Sci. 2022;79(6):1902–17. 10.1093/icesjms/fsac127.

[46] Waples RS. Genetic estimates of contemporary effective population size: to what time periods do the estimates apply? Mol Ecol. 2005;14(11):3335–52. 10.1111/j.1365-294X.2005.02673.x. 16156807 10.1111/j.1365-294X.2005.02673.x

[47] Waples RS, Do C. LDNE: A program for estimating effective population size from data on linkage disequilibrium. Mol Ecol Resour. 2008;8(4):753–6. 10.1111/j.1755-0998.2007.02061.x. 21585883 10.1111/j.1755-0998.2007.02061.x

[48] Waples RS, Do C. Linkage disequilibrium estimates of contemporary Ne using highly variable genetic markers: a largely untapped resource for applied conservation and evolution. Evol Appl. 2010;3(3):244–62. 10.1111/j.1752-4571.2009.00104.x. 25567922 10.1111/j.1752-4571.2009.00104.x PMC3352464

[49] Weir BS, Cockerham C. Estimating F-statistics for the analysis of population structure. Evolution. 1984;38(6):1358–70. 10.2307/2408641. 28563791 10.1111/j.1558-5646.1984.tb05657.x

[50] Weise EM, Van Wyngaarden M, Den Heyer C, Mills Flemming J, Kess T, Einfeldt AL, Fisher JAD, et al. SNP Panel and Genomic Sex Identification in Atlantic Halibut (*Hippoglossus hippoglossus*). Mar Biotechnol. 2023;25(4):580–7. 10.1007/s10126-023-10227-2. 10.1007/s10126-023-10227-2 37351707

[51] Westgaard J-I, Saha A, Kent M, Hansen HH, Knutsen H, Hauser L, Cadrin SX, et al. Genetic population structure in Greenland halibut (*Reinhardtius hippoglossoides*) and its relevance to fishery management. Can J Fish Aquat Sci. 2017;74(4):475–85. 10.1139/cjfas-2015-0430.

