# Supplementary material for: Genetic divergence of farmed Atlantic halibut (Hippoglossus hippoglossus) and potential for impact on wild populations
Source: BMC Res Notes. 2026 Apr 10;19:227. doi: 10.1186/s13104-026-07806-6 (PMC13185307; doi:10.1186/s13104-026-07806-6)
Supplement: Supplementary file 1 — Supplementary Material 1. [file 13104_2026_7806_MOESM1_ESM.docx]

**SUPPLEMENT**

Genetic divergence of farmed Atlantic halibut (*Hippoglossus hippoglossus*) and potential for impact on wild populations
Solveig Tronsgaard Rasmussen^1^, María Quintela^2*^, Mikko Vihtakari^3^, Geir Dahle^2^, François Besnier^2^, Ian Bradbury^4,5^, Michael Møller Hansen^1^, Kevin A. Glover^2^, Per Erik Jorde^6^

**Fig. S1.** Positions of 96 SNPs mapped to the halibut genome (GenBank assembly GCA_009819705.1). Vertical blue bars: chromosome outlines with the sex-associated region (Einfeldt et al. 2021, p.1690) on chromosome 12 coloured orange.


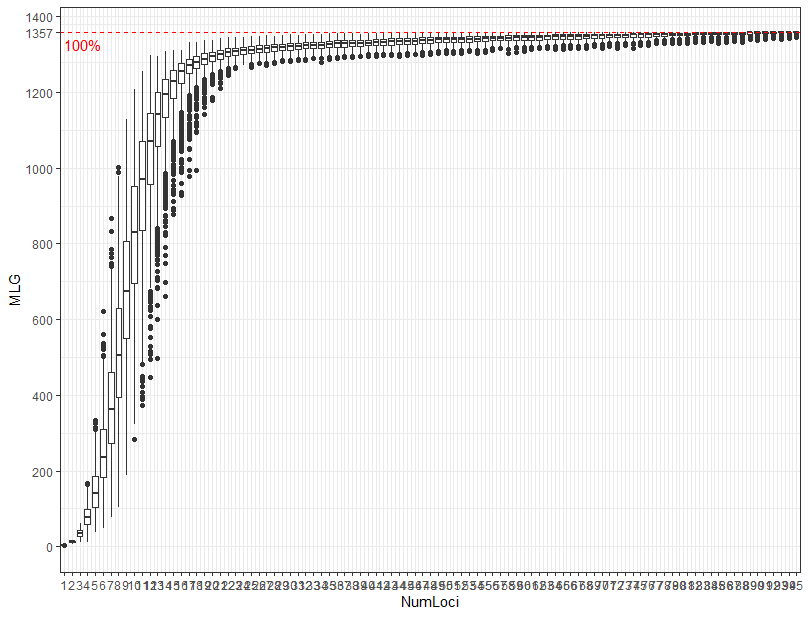


**Fig. S2.** Genotype accumulation curve calculated for the set of 96 SNP loci using the total 1368 individuals.

**
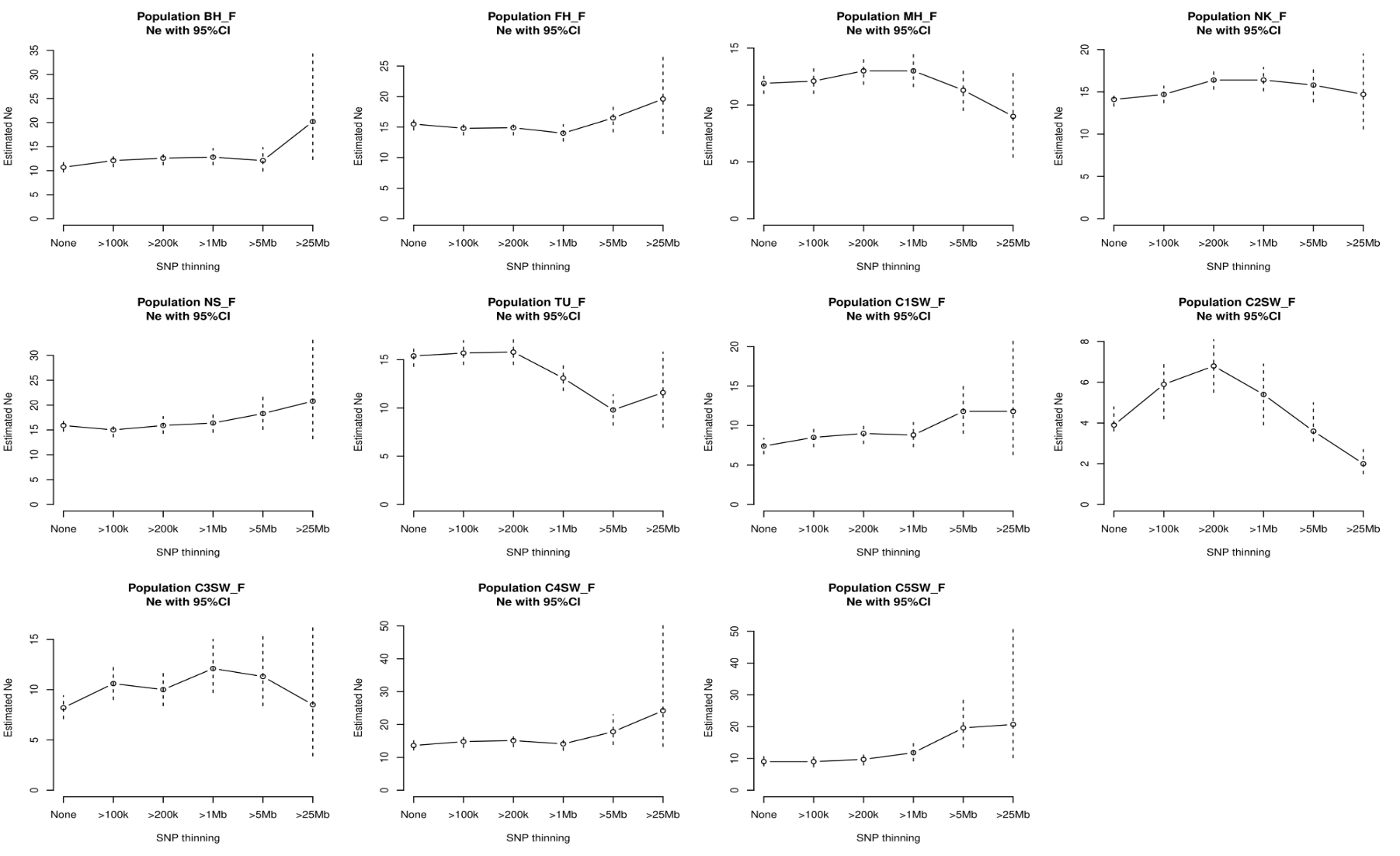
**

**Fig. S3.** Evaluation of filtering (thinning) of SNPs on estimated effective size of farmed halibut populations. SNPs were thinned on basis of their physical distances, from left to right within each panel: no thinning (all 111 SNPs); >100Kb separation between SNPs (96 SNPs); >200Kb (79 SNPs); >1Mb (67 SNPs); >5Mb (43 SNPs); and >25Mb (i.e. on different chromosomes: 19 SNPs). Dotted vertical bars: 95% confidence intervals. All estimates used the LDNe software v1.31 (Waples & Do 2008).


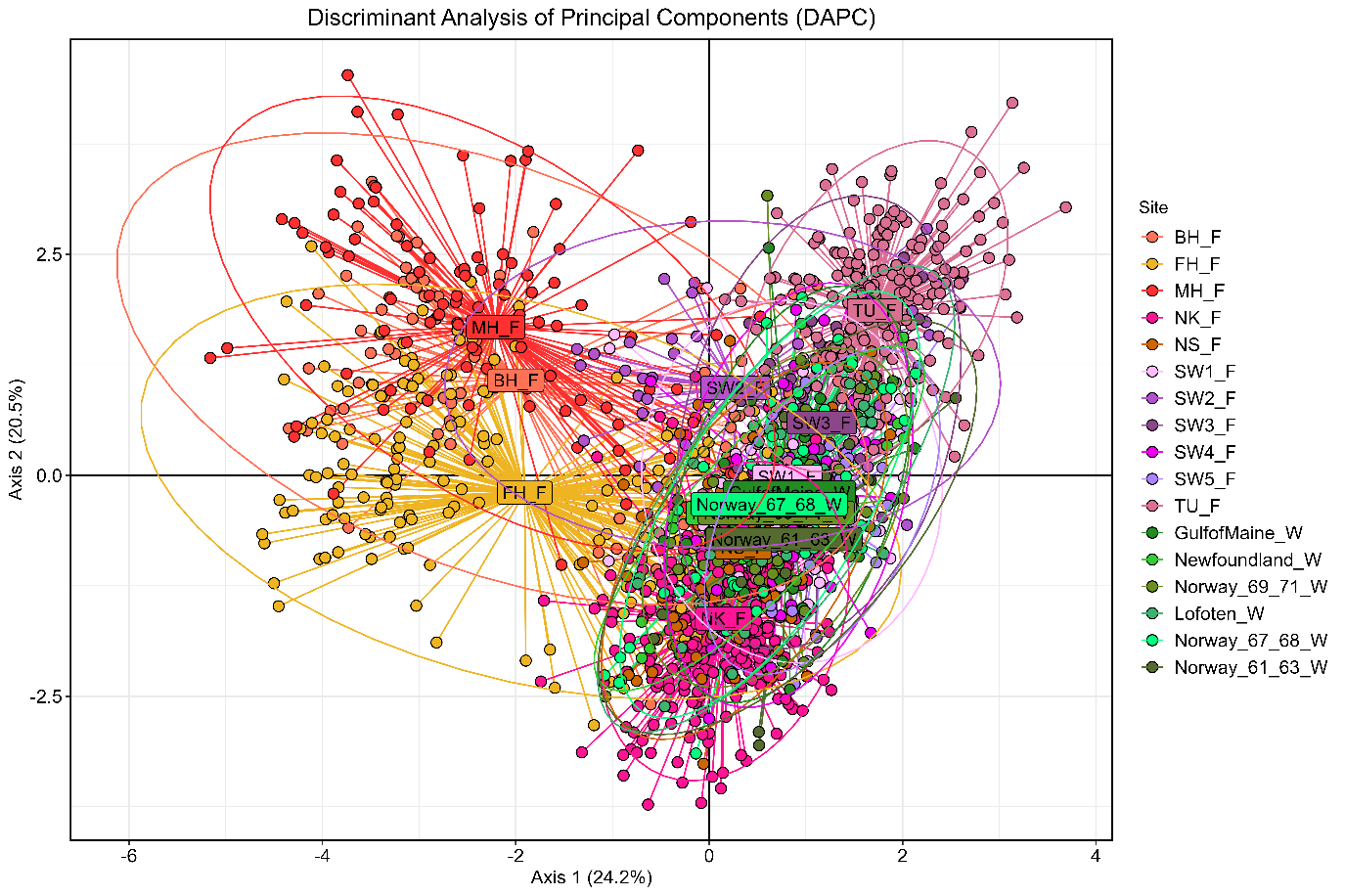


**Fig. S4.** Discriminant Analysis of Principal Components (DAPC) of Atlantic halibut based upon 111 polymorphic SNP loci. The axes correspond to the two first discriminant functions after retaining 90 principal components. Individuals from different geographically explicit samples are represented by coloured dots, and name labels are centred on the mean of the inertia ellipse. Greenish colours depict the wild individuals.

| a)  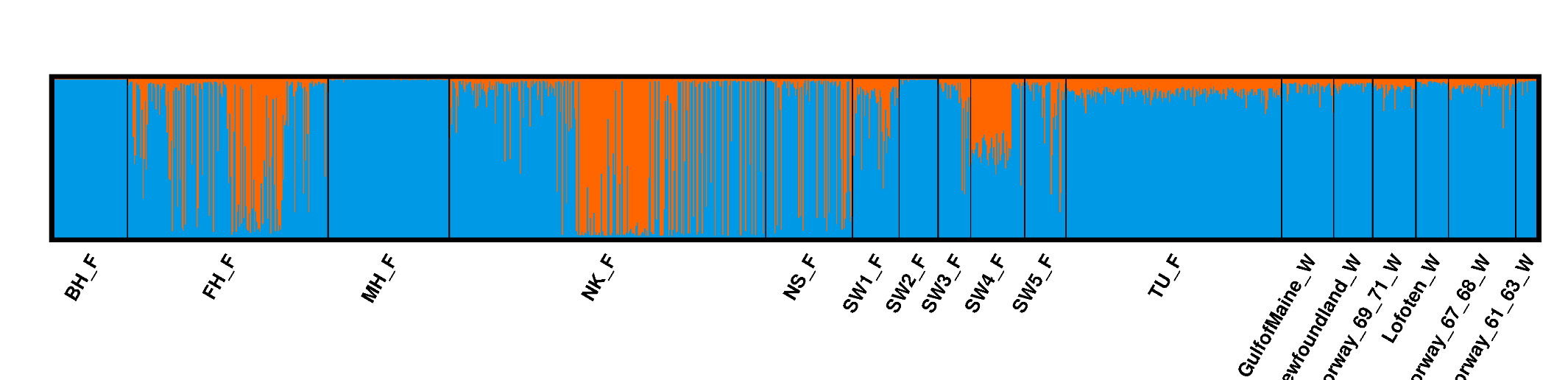 |
| --- |
| b)  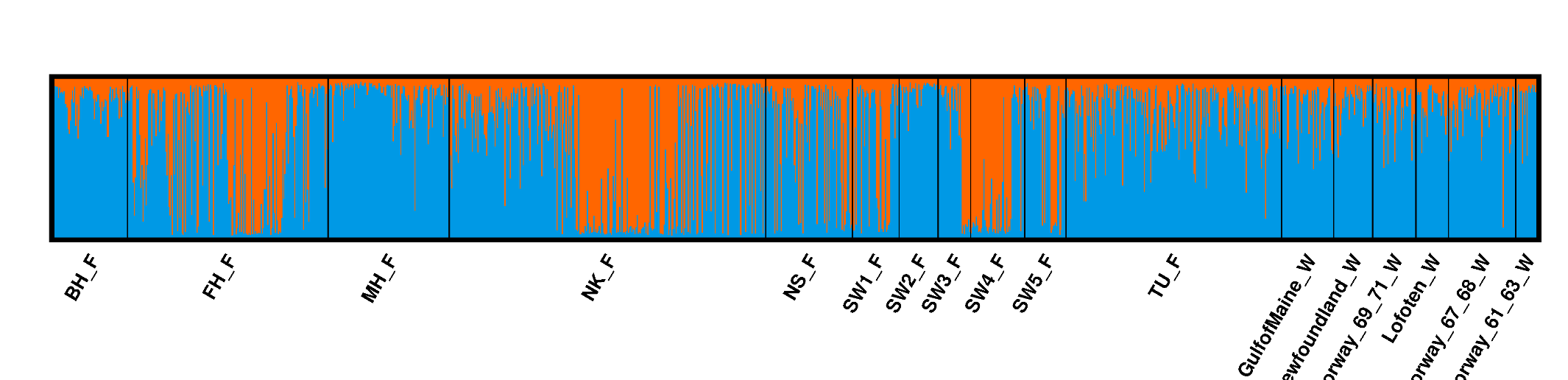 |

**Fig. S5.** Barplot representing the proportion of Atlantic halibut individuals’ ancestry to cluster at K2 after Bayesian clustering in STRUCTURE a) with and b) without LOCPRIORS.
